# Effectiveness and Safety of Interventions for Treating Adults with Displaced Proximal Humeral Fracture: A Network Meta-Analysis and Systematic Review

**DOI:** 10.1371/journal.pone.0166801

**Published:** 2016-11-18

**Authors:** Long Chen, Fei Xing, Zhou Xiang

**Affiliations:** 1 Department of Orthopedics, West China Hospital, Sichuan University, Chengdu, Sichuan, China; 2 Department of Orthopedics, Guizhou Provincial People's Hospital, Guiyang, Guizhou, China; Harvard Medical School/BIDMC, UNITED STATES

## Abstract

**Purpose:**

Network meta-analysis (NMA) is a comparatively new evidence-based technique in medical disciplines which compares the relative benefits associated with multiple interventions and obtains hierarchies of these interventions for various treatment options. We evaluated the effectiveness and safety of open reduction and internal fixation (ORIF), hemiarthroplasty (HA), reverse shoulder arthroplasty (RSA), intramedullary nailing (IN) and non-operative treatment (NOT) of displaced proximal humeral fractures in adults using Bayesian NMA of data from clinical trials.

**Method:**

PUBMED, EMBASE and CENTRAL in July 2016 were searched and clinical trials that evaluated interventions for treating adults with displaced proximal humeral fractures were identified. Methodological qualities of studies were assessed by the Newcastle—Ottawa Scale and risk of bias using the Cochrane Collaboration tool.

**Result:**

Thirty-four trials involving 2165 participants were included in the study. RSA had significantly the highest Constant score and lower total incidence of complications than ORIF, HA and IN. Moreover, RSA resulted in a lower incidence of additional surgery than ORIF and IN. The rank of treatments in terms high Constant score was: RSA, ORIF, IN, NOT and HA. The rank for reduction in total incidence of complications was: RSA, NOT, HA, IN and ORIF. For lowering the risk of additional surgery, the rank was: RSA, NOT, HA, IN and ORIF.

**Conclusion:**

RSA had the highest probability for improving functional outcome and reduction in the total incidence of complications and requiring additional surgery among the five interventions for treating adults with displaced proximal humeral fracture.

## Introduction

Fractures of the proximal humerus are the third most common in elderly patients after those of the hip and distal radius [1], accounting for 5% to 6% of all adult fractures [2]. Their incidence rapidly increases with age, and women are affected between two and three times as often as men [2–4]. Both non-operative and operative methods are used to treat these fractures. Non-operative treatment (NOT) involves a period of immobilization, such as an arm sling, followed by physiotherapy and exercise. It is generally the accepted treatment option for minimally displaced fractures and often used also for people with displaced fractures [3]. Operative treatment is recommended for displaced and unstable fractures and those with more complex fracture patterns to avoid painful and dysfunctional malunion [5]. The common operative treatments include open reduction and internal fixation (ORIF), hemiarthroplasty (HA), reverse shoulder arthroplasty (RSA), intramedullary nailing (IN). The debate among surgeons regarding which treatment should be used is still unresolved today and has been since the 1980s. Previous pairwise meta-analyses [3, 5–8] could not obtain hierarchies of these treatments because some had not been compared one by one.

We compared the effectiveness and safety of these five treatments (NOT, ORIF, HA, RSA, IN) for displaced proximal humeral fracture in adults by network meta-analysis (NMA). Our intention was to provide hierarchies of the comparative Constant score, total incidence of complications and need for additional surgery.

NMA is a powerful technique that has been used for more than a decade to rank treatment options with both direct comparisons of treatments in randomized controlled trials and indirect comparisons across trials with a common comparator. We ensured validity through careful review design and rigorous analysis of criteria for inclusion of the various studies.

## Methods

### Eligibility criteria and literature search

This study was performed in accordance with the Preferred Reporting Items for Systematic Reviews and Meta-Analyses (PRISMA) statement [9]. We searched the Cochrane Register of Controlled Trials (CENTRAL, Jul 2016), PubMed (Jan 1980 to Jul 2016), and EMBASE (1980 to Jul 2016) databases to identify all studies that discussed the effectiveness and safety of NOT, ORIF, HA, RSA, IN. Keywords and MeSH terms used in the search strategy included “proximal humeral fracture”, “non-operative treatment”, “open reduction and internal fixation”, “hemiarthroplasty”, “reverse shoulder arthroplasty” and “intramedullary nailing”.

The inclusion criteria were: (1) target population: patient was aged 16 years or older and presented within 3 weeks after sustaining a displaced fracture of the proximal humerus; (2) intervention: NOT, ORIF, HA, RSA, IN; (3) methodological criteria: randomized controlled trials and clinical trials. The exclusion criteria were: (1) target population: patient was aged under 16 years or presented more than 3 weeks after sustaining a displaced proximal humeral fracture; (2) methodological criteria: case reports and cohort studies. The study selection was conducted by two independent reviewers. Any disagreement between review authors was resolved by discussion.

### Outcome assessment

The primary outcome measure was Constant score [10] (activity, mobility, strength and pain). The secondary outcome measures included total complications (*e*.*g*. surgical site infection, symptomatic malunion, transient paresthesia and avascular necrosis of the humeral head) and incidence of additional surgery.

### Data extraction and quality assessments

Study type, country, sample size, length of follow-up and interventions data were gathered from each trial. Data on random sequence generation, allocation concealment, blinding, selective reporting and incomplete outcome data were gathered from randomized controlled trials. Data on representativeness of cases, selection of controls, definition of controls, comparability of cases and controls, ascertainment of exposure, equivalent methods of diagnosis and determination of response rate for cases and controls were gathered from controlled clinical trials. In addition, the following clinical data were extracted if available: Constant score, total number of complications and incidence of additional surgery.

The Cochrane Collaboration tool for assessing risk of bias [11] was used to assess the quality of randomized controlled trials, and the Newcastle—Ottawa Scale [12] was used to assess the quality of case—control trials in terms of selection and comparability of the study groups and determination of outcomes. In evaluating randomized controlled trials by the Cochrane Collaboration tool, quality of the studies was assessed using the following criteria: (1) randomization sequence generation: assessment for selection bias; (2) allocation concealment: assessment of selection bias; (3) level of blinding (blinding of participants and blinding of outcome assessment): assessment for performance bias and detection bias; (4) incomplete outcome data: assessment for attrition bias; and (5) selective reporting: assessment for reporting bias [11]. For case—control studies, the total Newcastle—Ottawa Scale score was calculated with a maximum of nine points using the criteria listed in S1 Table [12].

### Data synthesis and analysis

Two researchers extracted data independently according to the prespecified selection criteria. Disagreements were resolved by discussion. In each study, the relative risk (RR) was calculated for dichotomous outcomes (*e*.*g*. total complications and incidence of additional surgery), and treatment effects for continuous outcomes (*e*.*g*. Constant score) including mean differences (MDs) for studies with comparable outcome measures used a 95% confidence interval (CI).

We performed conventional pairwise meta- analyses for all outcomes and comparisons, using a random effects model by STATA (version 12.0, Stata Corp, College Station, TX). The pooled estimates of RRs or standardized MDs and 95% CI of three outcomes (Constant score, total incidence of complication and additional surgery) were shown. NMA combines direct and indirect evidence within a Bayesian framework and was implemented using WinBUGS statistical software (version 1.4.3) using Markov Chain Monte Carlo (MCMC) methods. The models, codes and software used in this study are available free online [13]. We performed surface under the cumulative ranking curve (SUCRA) probabilities to rank the five interventions for treating displaced proximal humeral fractures [14]. SUCRA is a proportion, expressed as a percentage of the efficacy of an intervention on the outcome that would be ranked first without uncertainty, and so equals 100% when the treatment is certain to be the best and 0% when certain to be the worst [14].

Inconsistencies of this NMA were assessed by the Higgins model. Significance levels smaller than 0.05 were interpreted as evidence of inconsistency. The sensitivity analysis was performed by comparing the results of different effects models (random effects and fixed effects model).

## Results

### Study selection

**Fig 1:**
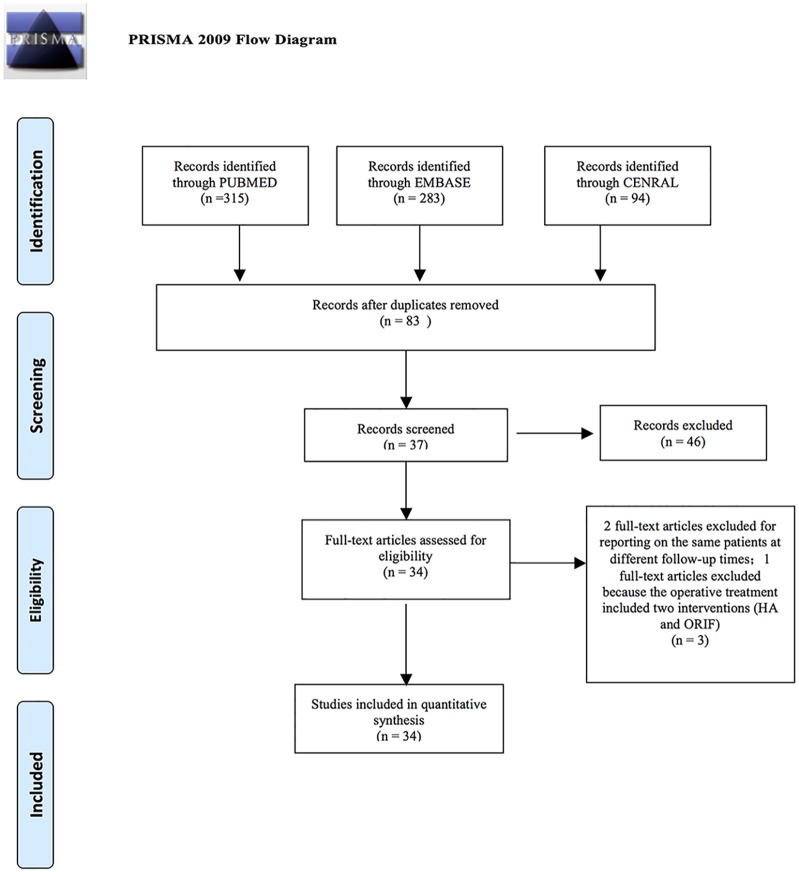
Flow chart of selection of studies for inclusion in meta- analysis. *From*: Moher D, Liberati A, TetZlaff J, Altman DG, The PRISMA Group (2009). *P*referred *R*eporting *I*tems for *S*ystematic Reviews and *M*eta-*A*nalyses: The PRISMA Statement. PLoS Med 6(7): e1000097. doi: 10.1371/journal.pmed1000097 **For more information, visit** www.prisma-statement.org.

Fig 1 shows the study selection process according to the PRISMA statement. This search strategy retrieved a total of 692 studies: 94 studies were from CENTRAL, 315 studies from PUBMED and 283 studies from EMBASE. Titles and abstracts of these references were examined by two reviewers, and 34 studies [15–48] were identified for further analysis. One study [49] was excluded because the operative treatment included two interventions (HA and ORIF). We also found that Fjalestad (2010) [50], Fjalestad (2012) [51] and Fjalestad (2014) [24] reported on the same patients at different follow-up times, so we included only one article of the multiple studies. Ten randomized controlled trials and twenty four controlled clinical trials remained and were considered for primary relevant studies, which were all included in this NMA.

### Study characteristics and risk of bias in studies included in the review

Table 1 provides a summary of the studies in the review. A total of 2165 participants (mean age: 69 years) were included. For each intervention, 748 patients (mean age: 66.06 years) were assigned to ORIF therapy, 803 (mean age: 71.3 years) to HA therapy, 191 (mean age: 77.3 years) to RSA therapy, 267 (mean age: 61.8 years) to IN therapy and 153 (mean age: 73.9 years) to NOT therapy. Study sample size ranged from 18 to 368. All 34 studies directly compared one treatment with another. These studies were published between 1984 and 2014. Twenty four studies reported Constant score as an outcome. Thirty one studies used total complications as an outcome and twenty studies reported incidence of additional surgery as an outcome.

**Table 1:** Characteristics of Included Studies Comparing Different treatments for displaced proximal humeral fracture.

Study	Country	Interventions	Sample size (mean age: y)	Follow-up (month)	Study design	For analysis
Dietrich 2008	Germany	ORIF vs HA	52(82)/59(80)	12	RC	Constant score; Total complications; Incidence of additional surgeries
Bastian 2009	Switzerland	ORIF vs HA	44(60)/33(60)	60	PC	Constant score; Total complications; Incidence of additional surgeries
Solberg 2009	USA	ORIF vs HA	38(66.5)/48(67.4)	36	RC	Constant score; Total complications; Incidence of additional surgeries
Wang 2009	China	ORIF vs HA	12(49)/10(49)	20	RC	Constant score; Total complications
Zhang 2010	China	ORIF vs HA	28(66.5)/30(68.9)	28	RC	Constant score; Total complications
Kim 2011	Korea	ORIF vs HA	38(64.9)/26(64.9)	24	RC	Constant score; Total complications; Incidence of additional surgeries
Wild 2011	USA	ORIF vs HA	42(56.9)/15(66.4)	35	RC	Constant score; Total complications; Incidence of additional surgeries
Spross 2011	Switzerland	ORIF vs HA	22(75)/22(76)	30	RC	Constant score; Total complications; Incidence of additional surgeries
Cai 2012	China	ORIF vs HA	13(72)/19(72)	24	RCT	Constant score
Lu 2012	China	ORIF vs HA	26(67)/22(67)	6	RC	Constant score; Total complications
Gallinet 2009	France	HA vs RSA	17(74)/16(74)	14.5	RC	Constant score; Total complications
Young 2010	New Zealand	HA vs RSA	10(75.5)/10(77.2)	33	RC	Total complications
Garrigues 2012	USA	HA vs RSA	9(69.3)/10(80.5)	43.2	RC	Total complications
Boyle 2013	New Zealand	HA vs RSA	313(71.9)/55(79.6)	12	RC	Incidence of additional surgeries
Cuff 2013	USA	HA vs RSA	23(74.4)/24(74.4)	24	PC	Total complications; Incidence of additional surgeries
Fu 2013	China	HA vs RSA	12(69.5)/11(81.2)	43.2	RC	Total complications
Baudi 2014	Italy	HA vs RSA	28(71.4)/25(77.3)	27.5	RC	Constant score; Total complications
Chalmers 2014	USA	HA vs RSA	9(72)/9(77)	12	RC	Total complications
Sebastia-Forcada 2014	Spain	HA vs RSA	30(73.3)/31(74.7)	28.5	RCT	Constant score; Total complications; Incidence of additional surgeries
Gradl 2009	Germany	ORIF vs IN	76(63)/76(62)	12	PC	Constant score; Total complications; Incidence of additional surgeries
Matziolis 2010	Germany	ORIF vs IN	11(54.8)/11(55.6)	36	RC	Constant score; Total complications; Incidence of additional surgeries
Smejkal 2011	Chech	ORIF vs IN	28(61)/27(61)	2–18	RCT	Constant score; Total complications
Trepat 2011	Spain	ORIF vs IN	14(68.3)/15(64.5)	6–12	RC	Total complications; Incidence of additional surgeries
Zhu 2011	China	ORIF vs IN	26(50.5)/25(54.8)	12–36	RCT	Constant score; Total complications; Incidence of additional surgeries
Lekic 2012	USA	ORIF vs IN	12(59)/11(60)	3–46	RC	Total complications; Incidence of additional surgeries
Konrad 2012	Switzerland	ORIF vs IN	153(65.4)/58(64.8)	3–12	PC	Constant score; Total complications; Incidence of additional surgeries
Von 2014	Germany	ORIF vs IN	28(61)/44(61)	38–82	RC	Total complications; Incidence of additional surgeries
Zyto 1997	Sweden	ORIF vs NOT	20(73)/20(75)	36–60	RCT	Constant score; Total complications
Olerud 2011a	Sweden	ORIF vs NOT	30(74)/30(74)	24	RCT	Constant score; Total complications; Incidence of additional surgeries
Kollig 2003	Germany	ORIF vs NOT	13(52.5)/ 9(52.7)	74–82	PC	Constant score
Fjalestad 2014	Norway	ORIF vs NOT	25(72.2)/25(73.1)	24	RCT	Constant score; Total complications; Incidence of additional surgeries
Stableforth 1984	England	HA vs NOT	16(65.6)/16(70.1)	6–48	RCT	Total complications
Olerud 2011b	Sweden	HA vs NOT	27(75.8)/28(77.5)	24	RCT	Constant score; Total complications; Incidence of additional surgeries
Boons 2012	Netherlands	HA vs NOT	25(76.4)/25(79.9)	24	RCT	Constant score; Total complications; Incidence of additional surgeries

ORIF: open reduction and internal fixation; HA: hemiarthroplasty; RSA: reverse shoulder arthroplasty; IN: intramedullary nailing; NOT: Non-operative treatment; RC: Retrospective comparative; PC: Prospective comparative; RCT: Randomised controlled trial; y: years.

**Fig 2:**
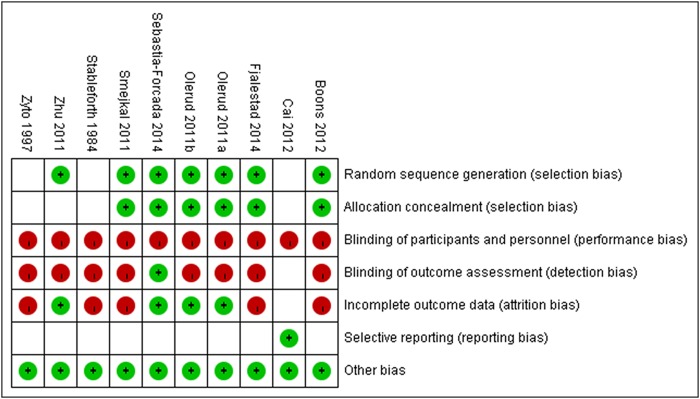
Risk of bias summary: review authors’ judgements about each risk of bias item for each included study.

Of the ten randomized controlled trials analyzed [17, 19, 24, 34–37, 40, 47, 48], the Cochrane Collaboration tool indicated that seven trials [17, 24, 34–37, 47] used adequate randomization and six trials [17, 24, 34–37] used adequate allocation concealment. One study [36] reported outcome assessment blinding and also one study [19] was free of selective reporting. Four trials [23, 34–36, 47] mentioned incomplete outcome data reporting (Fig 2). As assessed by the Newcastle—Ottawa Scale, six case—control studies [21, 22, 27, 30, 38, 39] were awarded a score of nine points, eleven studies [15, 16, 20, 25, 28, 29, 31, 41, 42, 45, 46] received a score of eight points, six studies [18, 23, 26, 33, 43, 44] received a score of seven points, and only one study [32] received a score of six points (Table 2).

**Table 2:** Quality assessment of case—control studies comparing different treatments for displaced proximal humeral fracture using Newcastle—Ottawa Scale.

Author group		Selection			Comparability		Exposure	
	Adequat-e case definitio-n	Representativeness of the cases	Selectio-n of Control-s	Definitio-n of Controls	Comparability of cases and controls	Ascertainm-ent of exposure	Same method of ascertainmen-t	Non Response rate
Bastian 2009	1	1	1	1	1	1	1	1
Baudi 2014	1	1	1	1	1	1	1	1
Boyle 2013	1	1	1	1	-	1	1	1
Chalmers 2014	1	1	1	1	2	1	1	1
Cuff 2013	1	1	1	1	2	1	1	1
Dietrich 2008	1	1	1	1	1	1	1	-
Fu 2013	1	1	1	1	1	1	1	1
Gallinet 2009	1	1	1	1	1	1	1	1
Garrigues 2012	1	1	1	1	1	1	1	-
Gradl 2009	1	1	1	1	2	1	1	1
Kim 2011	1	1	1	1	1	1	1	1
Kollig 2003	1	1	1	1	1	1	1	1
Konrad 2012	1	1	1	1	2	1	1	1
Lekic 2012	1	1	1	1	1	1	1	1
Lu 2012	1	1	1	1	-	1	1	-
Matziolis 2010	1	1	1	1	1	1	1	-
Solberg 2009	1	1	1	1	2	1	1	1
Spross 2011	1	1	1	1	2	1	1	1
Trepat 2011	1	1	1	1	1	1	1	1
Von 2014	1	1	1	1	1	1	1	1
Wang 2009	1	1	1	1	-	1	1	1
Wild 2011	1	1	1	1	1	1	1	-
Young 2010	1	1	1	1	1	1	1	1
Zhang 2010	1	1	1	1	1	1	1	1

### Constant score

For the primary outcome, 24 trials were included in the NMA. The following interventions for treating displaced proximal humeral fracture were tested in the trials: ORIF versus HA (10 trials with 533 patients) [15, 19, 23, 28, 32, 38, 39, 43, 44, 46]; HA versus RSA (3 trials with 147 patients) [16, 25, 36]; HA versus NOT (2 trials with 96 patients) [17, 34]; ORIF versus IN (5 trials with 491 patients) [27, 30, 33, 37, 47]; ORIF versus NOT (4 trials with 152 patients) [24, 29, 35, 48].

**Fig 3:**
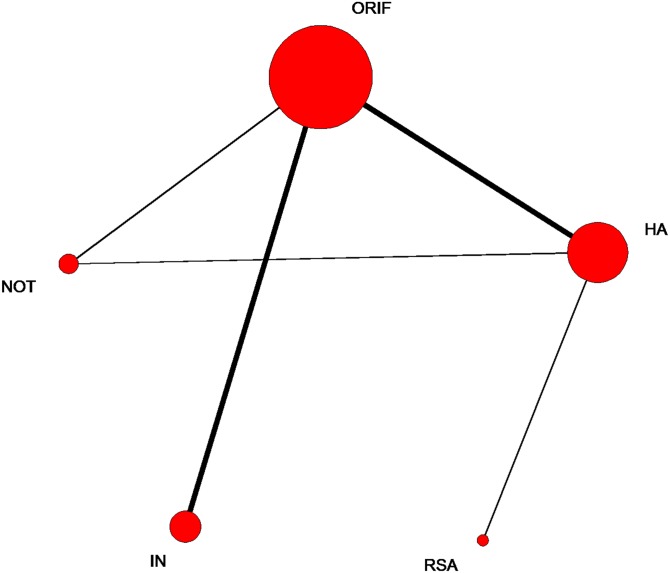
Network of treatment comparisons for constant score. The size of the node corresponds to the total sample size of treatments. Directly comparable treatments are linked with a line, the thickness of which represents the number of trials that were compared. ORIF: open reduction and internal fixation; HA: hemiarthroplasty; RSA: reverse shoulder arthroplasty; IN: intramedullary nailing; NOT: Non-operative treatment.

**Fig 4:**
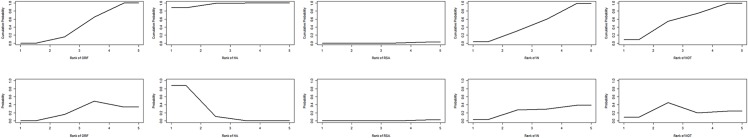
Ranking of treatment strategies based on the probability of their effects on the outcome of constant score. ORIF: open reduction and internal fixation; HA: hemiarthroplasty; RSA: reverse shoulder arthroplasty; IN: intramedullary nailing; NOT: Non-operative treatment.

The network of comparisons on Constant score is shown in Fig 3. Table 3 provides hierarchies of effect size on Constant score. Ranking graphs of the distribution of probabilities of Constant score are displayed in Fig 4. The direct and indirect comparisons indicated that HA significantly decreased the Constant score compared with the other groups and RSA was responsible for a significantly higher Constant score compared with the other groups. Based on SUCRA, HA ranked first (0.9675), the second was NOT (0.5905), IN was third (0.4805), the fourth was ORIF (0.4520) with RSA being last (0.0095).

**Table 3:** Results for constant score, from network meta-analysis (lower diagonal part) and pairwise meta-analysis (upper diagonal part).

**ORIF**	6.90(1.77–12.04)	NA	-0.175(-3.11–2.76)	-1.45(-7.12–4.22)
5.48(2.15–8.66)	**HA**	-14.68(-19.14–10.22)	NA	1.56(-5.48–8.60)
-9.15(-16.61–1.6)	-14.63(-21.26–8.11)	**RSA**	NA	NA
0.04(-5.17–5.17)	-5.44(-11.68–0.52)	9.19(0.44–18.13)	**IN**	NA
1.29(-4.45–7.08)	-4.19(-10.15–1.88)	10.44(1.82–19.49)	1.25(-6.16–9.68)	**NOT**

ORIF: open reduction and internal fixation; HA: hemiarthroplasty; RSA: reverse shoulder arthroplasty; IN: intramedullary nailing; NOT: Non-operative treatment

### Total incidence of complications

**Fig 5:**
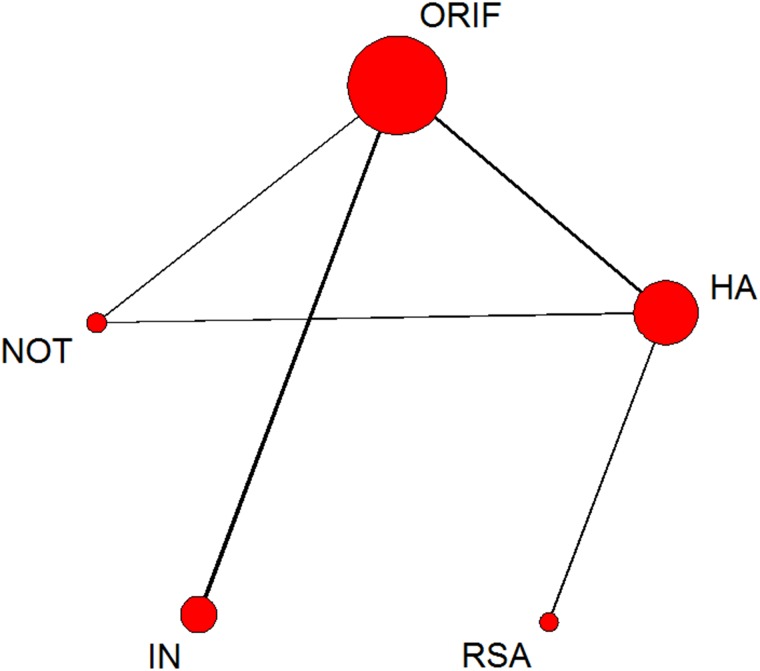
Network of treatment comparisons for incidence of total complications. The size of the node corresponds to the total sample size of treatments. Directly comparable treatments are linked with a line, the thickness of which represents the number of trials that were compared. ORIF: open reduction and internal fixation; HA: hemiarthroplasty; RSA: reverse shoulder arthroplasty; IN: intramedullary nailing; NOT: Non-operative treatment.

**Fig 6:**
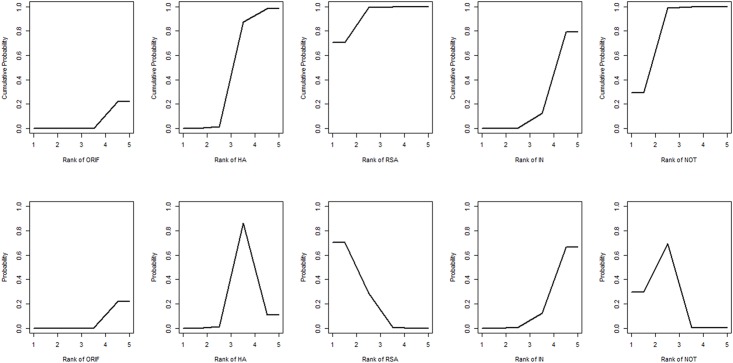
Ranking of treatment strategies based on the probability of their effects on the outcome of incidence of total complications. ORIF: open reduction and internal fixation; HA: hemiarthroplasty; RSA: reverse shoulder arthroplasty; IN: intramedullary nailing; NOT: Non-operative treatment.

For this outcome, 31 trials were included in the NMA. The network of comparisons on total incidence of complications is shown in Fig 5. Table 4 provides hierarchies of effect size on their incidence. The ranking graphs of distribution of probabilities of incidence can be seen in Fig 6. Direct and indirect comparisons indicate that RSA results in a lower incidence of complications than ORIF, HA and IN. Based on SUCRA, RSA ranked first (0.9248), second was NOT (0.8198), HA was third (0.4678), the fourth was IN (0.2318) with ORIF last (0.0560).

**Table 4:** Results for total complications, from network meta-analysis (lower diagonal part) and pairwise meta-analysis (upper diagonal part).

**ORIF**	1.35(0.95–1.92)	NA	1.08(0.76–1.53)	1.97(0.43–9.08)
1.95(1.08–3.16)	**HA**	2.35(1.18–4.67)	NA	1.52(0.86–2.70)
7.61(2.46–18.9)	3.93(1.44–8.8)	**RSA**	NA	NA
1.27(0.69–2.1)	0.7(0.31–1.38)	0.22(0.05–0.6)	**IN**	NA
5.18(2.1–10.22)	2.73(1.2–5.45)	0.86(0.23–2.5)	4.36(1.5–9.66)	**NOT**

ORIF: open reduction and internal fixation; HA: hemiarthroplasty; RSA: reverse shoulder arthroplasty; IN: intramedullary nailing; NOT: Non-operative treatment

### Incidence of additional surgery

**Fig 7:**
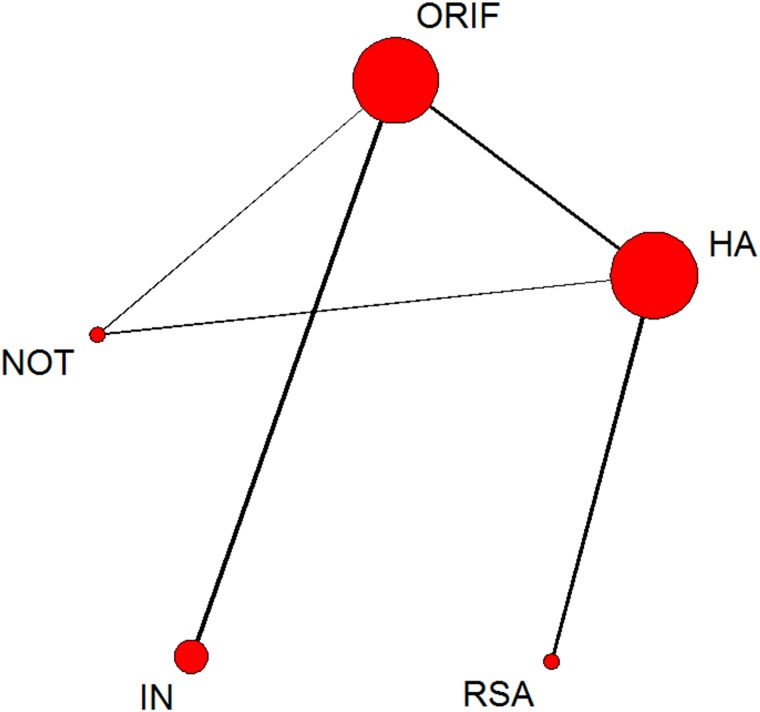
Network of treatment comparisons for incidence of additional surgery. The size of the node corresponds to the total sample size of treatments. Directly comparable treatments are linked with a line, the thickness of which represents the number of trials that were compared. ORIF: open reduction and internal fixation; HA: hemiarthroplasty; RSA: reverse shoulder arthroplasty; IN: intramedullary nailing; NOT: Non-operative treatment.

**Fig 8:**
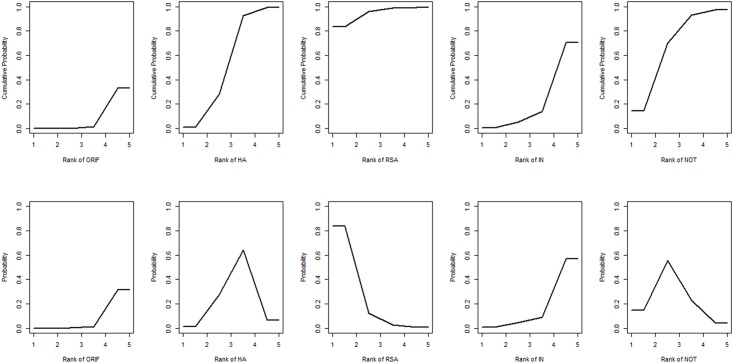
Ranking of treatment strategies based on the probability of their effects on the outcome of incidence of additional surgery. ORIF: open reduction and internal fixation; HA: hemiarthroplasty; RSA: reverse shoulder arthroplasty; IN: intramedullary nailing; NOT: Non-operative treatment.

For this outcome, 20 trials were included in the NMA. The network of comparisons on incidence of additional surgery is shown in Fig 7. Table 5 provides the hierarchies of effect size on incidence of additional surgery, with ranking graphs of the distribution of probabilities on incidence of additional surgery in Fig 8. Only indirect comparison indicates that RSA results in a lower incidence of additional surgery than ORIF and IN. Both direct and indirect comparisons suggest that HA results in a lower incidence of additional surgery than ORIF. Based on SUCRA, RSA ranks first (0.9450), NOT second (0.6870), the third was HA (0.5538), the fourth was IN (0.2272) with ORIF last (0.0870).

**Table 5:** Results for incidence of additional surgery, from network meta-analysis (lower diagonal part) and pairwise meta-analysis (upper diagonal part).

**ORIF**	2.15(1.14–6.13)	NA	0.98(0.56–1.69)	3.64(0.42–31.33)
6.14(1.18–24.74)	**HA**	2.89(0.62–13.44)	NA	1.98(0.36–10.97)
132.29(2.01–894.98)	19.22(0.75–119.46)	**RSA**	NA	NA
1.93(0.37–6.23)	0.5(0.04–2.08)	0.2(0–0.84)	**IN**	NA
13.91(0.81–65.2)	2.94(0.18–14.2)	0.98(0.01–5.45)	16.78(0.32–71.31)	**NOT**

ORIF: open reduction and internal fixation; HA: hemiarthroplasty; RSA: reverse shoulder arthroplasty; IN: intramedullary nailing; NOT: Non-operative treatment

### Inconsistency and sensitivity analysis

In general, the results obtained from the pairwise meta-analysis closely matched those of the NMA and no inconsistencies were identified in the NMA when using the Higgins model (Chi squared = 1.74, P = 0.1871 > 0.05). The sensitivity analysis was performed by comparing the results of different effects models (random effects and fixed effects models). The results of the random effects model (effective number of parameters [pD] = 38.7 and deviance information criterion [DIC] = 278.9) were similar to the fixed-effect model (pD = 28.1 and DIC = 322.9).

## Discussion

The NMA provided hierarchies for the Constant score, incidence of total complications and additional surgery in adult with displaced proximal humeral fracture treated with different methods, which had advantages over comparison with traditional pairwise meta-analyses [3, 5–8]. The meta-analysis indicated that: (1) HA significantly decreased Constant score compared with the other groups and RSA resulted in significantly higher Constant score compared with the other groups; (2) RSA resulted in a lower incidence of complications than ORIF, HA and IN; (3) RSA caused a lower incidence of additional surgery than ORIF and IN. (4) The rank of treatments in terms of Constant score was: RSA, ORIF, IN, NOT and HA; (5) For reduction in total incidence of complications, the rank of treatments was: RSA, NOT, HA, IN and ORIF; (6) The rank of treatments for lowering risk of additional surgery was: RSA, NOT, HA, IN and ORIF. RSA group included patients with higher mean age (77.3 years) and IN group included patients with lower mean age (61.8 years) than other treatments. A total of 2165 participants with the mean age of 69 years were included in this network meta-analysis. So the findings were suitable for the patients with mean age of more than 60 years.

There are some particular strengths to the analysis in this NMA: (1) It was conducted using common methods and was designed to allow for reproducible research selection and inclusion; (2) a broad and extensive search strategy was used to minimize the possibility of publication bias; (3) the study overcomes a major limitation of conventional pairwise meta-analysis by combining direct and indirect evidence of the efficacy of treatment strategies; (4) the SUCRA and posterior probabilities of outcomes were used to distinguish the subtle differences among five treatments.

However, this analysis has several limitations. Firstly, randomized controlled trials and case—control studies were both included in the analysis, and the case—control studies may have reduced the significance of the conclusions. Secondly, the lack of any treatment-provider blinded studies may have introduced detection bias, in which the assessors are likely to have preferentially attributed the occurrence of injury to the control group. Thirdly, some of the study characteristics such as type of fracture, age and performance bias might be potential obstacles to the outcomes of our study. Finally, these interventions (NOT, ORIF, HA, RSA and IN) may have different indications, so the comparisons between treatments within trials and sample for each trial may have interacted in ways that this analysis would not reveal. However, our network meta-analysis can still provide useful information about effectiveness and safety of interventions for treating adults with displaced proximal humeral fracture to the surgeons.

Xie [52] previously reported that operative treatments (including ORIF and HA) did not significantly improve Constant score and led to a higher incidence of postoperative complications compared with NOT. Li [6] also indicated that ORIF did not improve the Constant score when compared with NOT. Dai [5] showed that ORIF resulted in better Constant score than HA, and HA could reduce the rate of revisions and the method-related complications significantly. Wang [7] stated that treatment with ORIF caused no significant difference in Constant score and total number of complications compared with IN. Compared with HA, Shukla [53] reported that RSA resulted in more favorable Constant scores and Zhang [8] demonstrated that RSA was associated with a lower rate of total complications. Our network analysis showed that RSA resulted in significantly higher Constant score compared with the other four interventions. We also found that RSA resulted in lower total incidence of complications than ORIF, HA and IN. RSA also resulted in a lower incidence of additional surgery than ORIF and IN. However, we also use the SUCRA and posterior probabilities of outcomes to distinguish the subtle differences among five treatments. For achieving higher Constant scores the rank on treatments was: RSA, ORIF, IN, NOT and HA. For reducing incidence of total complications, the rank on treatments was: RSA, NOT, HA, IN and ORIF. For lowering risk of additional surgery, the rank on treatments was: RSA, NOT, HA, IN and ORIF.

## Conclusions

In summary, this Bayesian NMA of data from clinical trials demonstrated that RSA has the highest probability of improving the functional outcome and reducing the total incidence of complications and additional surgery among the five interventions for treating adults with displaced proximal humeral fracture.

## Supporting Information

S1 Table Newcastle-Ottawa Scale (NOS) for assessing the quality of case control studies in meta-analyses. (DOCX)

S2 Table PRISMA Checklist for this meta-analysis. (DOC)
