# Supplementary material for: Effectiveness and Safety of Interventions for Treating Adults with Displaced Proximal Humeral Fracture: A Network Meta-Analysis and Systematic Review
Source: PLoS One. 2016 Nov 18;11(11):e0166801. doi: 10.1371/journal.pone.0166801 (PMC5115806; doi:10.1371/journal.pone.0166801)
Supplement: S1 Table — (DOCX) [file pone.0166801.s001.docx]

| **Selection** | **Comparability** | **Exposure** |
| --- | --- | --- |
| 1) Is the case definition adequate? | 1) Comparability of cases and | 1) Ascertainment of exposure |
| a) yes, with independent validation* | controls on the basis of the design | a) secure record (eg: surgical records)* |
| b) yes, eg record linkage or based on self reports | or analysis | b) structured interview where blind to |
| c) no description | a) study controls for __ (Select | case/control status* |
| 2) Representativeness of the cases | the most important factor.)* | c) interview not blinded to case/control |
| a) consecutive or obviously representative series | b) study controls for any | status |
| of cases* | additional factor (This criteria | d) written self report or medical record |
| b) potential for selection biases or not stated | could be modified to indicate | only |
| 3) Selection of Controls | specific control for a second | e) no description |
| a) community controls* | important factor.)* | 2) Same method of ascertainment for cases |
| b) hospital controls |  | and controls |
| c) no description |  | a) yes* b) no |
| 4) Definition of Controls |  | 3) Non-Response rate |
| a) no history of disease (endpoint)* |  | a) same rate for both groups* |
| b) no description of source |  | b) non respondents described |
|  |  | c) rate different and no designation |
|  | | |
| Note: A study can be awarded a maximum of one star for each numbered item within the Selection and Exposure categories. A maximum of two stars can be given for Comparability. | | |

S1 Table Newcastle-Ottawa Scale (NOS) for assessing the quality of case control studies in meta-analyses

From GA. W, B. S, D. OC, et al. The Newcastle-Ottawa Scale (NOS) for assessing the quality of nonrandomised studies in meta-analyses.
